# Consultants and General Practitioners Debate

**Published:** 1906-12-08

**Authors:** 


### CONSULTANTS AND GENERAL PRACTITIONERS DEBATE.

### THE MISUSE OF HOSPITALS.

The first of a series of discussions on "Economic Problems in the Practice of Medicine," under the auspices of the Marylebone Division of the British Medical Association took place at the rooms of the Royal Medico-Chirurgical Society on Monday, when the question of gratuitous medical relief by the voluntary hospitals was under debate. The chair was taken by Dr. Lauriston Shaw.

The following resolution was submitted to the meeting :—

That it is desirable that concerted action should be taken to check the large amount of gratuitous medical attendance granted to many sections of the community, inasmuch as it tends to diminish the thrift and self-respect of the public, lessens the esteem in which medical science is held, and has a detrimental effect upon the position of many members of the medical profession.

Sir Richard Douglas Powell, Bart., opened the discussion, and confessed that he approached the subject with no little anxiety, as it was a matter which required very careful consideration from all its aspects. The more one looked into the question the greater seemed to be the difficulty of adjusting the relationship between institutions such as hospitals and the medical practitioners, and especially those who worked among that class for whom at the present time a considerable proportion of hospital patients was drawn. In a document on Hospital Reform which had been sent round to the large hospitals, containing suggested model principles of hospital management, the first principle laid down was that inability to pay for adequate treatment should be the test for the admission of patients to the hospitals. That at once raised the question, what was adequate treatment and how far were the lower-middle and artisan classes able to secure adequate treatment outside large institutions such as hospitals? Before considering that point the speaker laid down two propositions : First, that the original idea of the hospital was that it was for the reception and treatment of the indigent poor, but that model principle considerably enlarged the scope of that idea. Secondly, that the medical profesion was almost unique in two respects, first in that it had to deal with a commodity—health—which, however important to the individual, could not be said to have any money equivalent; and second, that the healing of the sick had never been a business, and that, as an outcome of its early associations with religion and religious bodies, any money equivalent for medical services had been more or less in the nature of an offering rather than an exact payment. Medicine being therefore a calling and not a business, still less a trade, they had to consider how they could harmonise that idea with a greater measure of appreciation and support for those who practised medicine as a profession. Within the memory of a great many of those present was the time when the mortality of the great hospitals was so large, and the administration so comparatively harsh, as to operate as a deterrent to those who might possibly seek hospital aid. Their poverty alone, and not their will, forced them to enter their doors. When in the order of things improvements in nursing, antiseptic methods of treatment, and the modern system of hospital hygiene were adopted, the mortality became so low, cases were so well treated and so humanely and gently handled, that patients' wills and not their poverty led them into the hospitals. So costly, too, had the treatment of many diseases—surgical especially, perhaps, but medical as well—become, that it was difficult for persons of small means, and yet above the indigent poor, to get adequate treatment except in some institution. If very costly operating theatres and appliances were really a necessity and not merely a counsel of perfection, then there was no way of meeting the difficulty of adequate treatment than some large conglomerate institutions for the reception of the lower-middle and artisan classes. They must either establish big provident hospitals or allow the present state of things to go on. It seemed to him, however, that if the principles, as distinguished from what one might call the ornaments, of aseptic and antiseptic medicine were to be thoroughly grasped by the patients who left the hospitals, an immense number of cases now sent to hospitals might be treated quite as well at home. For that, however, there were certain requisites, first a considerable staff of visiting or district nurses to prepare the rooms for minor operations, to nurse the patients who required skilled nursing, and to direct the nursing by friends of the patients who could be so cared for. Next, a depot or depots where dressings and appliances could be obtained at a reasonable rate; and thirdly, stores where drugs could be obtained at similar reasonable rates. These three essentials could only be secured by co-operation, by some combination between the private dispensary and sick assurance societies, and it was evident that organisation and education were necessary before the profession could say that patients of the classes under consideration could be adequately treated at home. As regards the out-patient class, properly organised provident dispensaries where advice and remedies could be obtained at adequate fees which would secure for every patient thoroughness and care in treatment, were necessary. If these centres were established, he thought that the out-patient departments could be much more restricted. These dispensaries might in some way be associated in a consultative capacity with the physicians and surgeons of the great hospitals. In addition to all this, the public would have to be educated to the necessity and the duty of contributing a fraction of their earnings during health for adequate medical treatment during sickness. All this meant very great difficulties and much consideration of detail, but he thought that if some strong committee of that association were to take the matter in hand and thoroughly deliberate upon it, some scheme might yet be evolved which would be the first step in the concerted action that was so desirable to lessen the large amount of gratuitous medical attendance granted to many sections of the community.

Sir John Tweedy prefaced his remarks with the proposition that free medical attendance came within the category of charitable relief. There was very little difference to his mind between almsgiving and giving medical relief. There was, however, this difference between ordinary poverty and sickness. Ordinary poverty, in a rightly constituted mind, stimulated the activity, whereas sickness was incapacitating and so led to actual poverty. The duty of the community was therefore to relieve the sick as quickly as possible and to administer their charities in such a way that they did the maximum of good to the sufferer, and were free, as far as practicable, from that evil to which they were universally found to be liable to tend—namely, indiscriminate charitable relief. Dealing with the practical ethical side of this question, he submitted that what was in the minds of most of them was that the hospital system, as at present administered, did tend to foster many of the evils which led to indiscriminate almsgiving. The hospital system as at present conducted accomplished a great work, but it also did no small amount of harm both to the patients and also to the profession. They all knew that there was a large proportion of the community who lived just above the level of poverty, and in that class sickness was more incapacitating than in the stratum just above that, and it was that class of society that the voluntary hospitals was intended to relieve promptly. The pauper was already provided for by the State and did not come within the category of the patients to be treated by the voluntary hospitals. His own belief was that there was, in the common acceptation of the term, very little hospital abuse so-called, but there was a great deal of hospital misuse, and that was the evil which they ought to attempt to adjust. He admitted that large numbers of patients were taken into the hospitals of London and into the out-patient departments for compulsory gratuitous medical relief who ought not to be so admitted, but who should receive treatment in connection with the great medical charities at some rate of payment. In conclusion, whatever plan was ultimately proposed or agreed upon to remedy the evil, he submitted that the following were acknowledged facts: that the evil existed, that it was increasing, and that some steps should be taken to check it, always bearing in mind that there was and must be in large towns like London, a large proportion of persons just above the pauper class, respectable people, for whom it was their duty and privilege to make provision in sickness; and that, on the other hand, there was above that class the well-to-do artisan, a good-wage-earning class, who were also entitled to help. So that whilst doing all they could to check the evils of indiscriminate medical relief, they should at the same time encourage all sections of the community above the pauper class to make complete provision for ordinary illness.

Dr. J. Kingston Fowler limited himself to one side of that many-sided question—the out-patient question. During the last twenty or twenty-five years that question of attendance at the out-patient department of the hospitals had been ever with them, and no progress had been made in remedying it, in fact he thought the position was worse now than it was twenty years ago. Why was this? The first reason was that the managers of our hospitals were in competition for patients one with another. They had to show, in order to attract the charitable, that they were doing a great work. The next reason was that the medical staff themselves had stood in the way of reducing the out-patient department on the plea that a large number of patients was necessary for the purposes of medical education. At one time that might have been true, but now it was not so. The greater number of ordinary cases were not used for the purposes of medical instruction. Next, it had always been proposed that the question should be settled by the joint action of the hospitals—a combination which there was not much chance of bringing about. Lastly, many persons held that the cure would be found in the establishment of provident dispensaries, but it would be perfectly impossible to do this so long as the doors of the out-patient departments were as widely open as they now were. The remedy he would suggest for the reduction of the out-patient department was concerted action on the part of King Edward's Hospital Fund and the Hospital Sunday and Saturday Funds, who could exert upon the hospitals the only power which would produce any effect —the power of the purse. The result would be that as the out-patient departments were closed, provident dispensaries would have to be opened, and eventually there would arrive the time when every person presenting himself at the out-patient department of a hospital would have to satisfy the officers there that he had been too poor to belong to the provident dispensary in his own district. A system on these lines was working successfully at Northampton.

Dr. William Hunter thought one solution of the question would be the establishment of a series of small homes so that patients who required operations or medical attendance in serious illnesses could be taken in there under their own doctors at reasonable rates.

Mr. Herbert Tanner held that hospitable abuse did exist, and instanced in confirmation of that the evidence which had been placed before him within the last two or three weeks as Chairman of one of the divisions of that Association.

Dr. Heron thought a remedy was to be found in the establishment of a system of insurance. In Germany at the present time they had established insurance societies on three lines—(1) Insurance against sickness, (2) insurance against accidents, and (3) insurance for old-age pensions and for payments to people who were chronic invalids. Towards the first-named the workers contributed two-thirds of the funds and the employers one-third; towards the second the funds came from the employers only; and in the third case the funds were equally contributed by the employers and the workers. In the result, in the year 1903, the last year for which statistics were available, the gross capital of the three systems of insurance amounted to £73,150,000, whilst the expenditure during that year was £23,550,000.

Dr. Kaye, who described himself as one of the rank and file of the medical profession, practising amongst the lower-middle classes in what was practically an East-end district, held that the general practitioner could not afford to treat this question of hospital abuse in an aloof manner. It affected not only themselves but also the class amongst whom they practised—a class who were being decidedly demoralised by it. They had been told that evening that there was no hospital abuse; but was there a medical man in London doing an average practice who did not every day in the year see patients in his surgery who were perfectly able to pay for their medical treatment who told him that they had been to this hospital or to that hospital?

Dr. Hawthorne appealed to his hearers for an impartial consideration of the subject and hoped that the medical profession would be careful not to put in the forefront of their discussions anything which might be considered to be their commercial interests.

Other speakers followed and the resolution, on being put to the meeting, was carried, with one dissentient.

*** Owing to great pressure on our space, we have been obliged to hold over our report of the Royal Free Hospital festival dinner and other matters.—Ed. The Hospital.

